# Special Issue: “Plant Virus Pathogenesis and Disease Control”

**DOI:** 10.3390/v12091049

**Published:** 2020-09-21

**Authors:** Bryce W. Falk, Shahideh Nouri

**Affiliations:** 1 Department of Plant Pathology, University of California, Davis, CA 95616, USA; 2 Department of Plant Pathology, Kansas State University, Manhattan, KS 66506, USA

Plant viruses are emerging and re-emerging to cause important diseases in many plants that humans grow for food and/or fiber, and sustainable, effective strategies for controlling many plant virus diseases remain unavailable. However, cutting-edge technological approaches in cell and molecular biology and rapidly accumulating virus sequence datasets are allowing us to gain new insights into mechanisms involved in pathogenesis and how interactions between plants and viruses affect disease development. In some cases, these offer opportunities for novel approaches to target plant viruses and help control the diseases they cause. Seven original articles in this Special Issue of *Viruses* on “Plant Virus Pathogenesis and Disease Control” give new, important information addressing different aspects of this topic.

Tomato brown rugose fruit virus (TBRFV) is a recently described and rapidly spreading tobamovirus affecting tomatoes (*Solanum lycopersicum*) in several parts of the world. Klap and colleagues show further complexity associated with TBRFV-induced disease. They show that when TBRFV co-infects plants with the potexvirus, pepino mosaic virus (PepMV), more severe symptoms develop [1]. PepMV can be an important pathogen in its own right, but PepMV mild strains are not uncommon and some are even used in disease control by cross-protection. Klap and colleagues show that when mild PepMV strains co-infect plants with TBRFV, the PepMV titer increases in doubly-infected plants and a more severe disease resembling that caused by aggressive PepMV strains is the result.

Soybeans (*Glycine max*) are a globally important crop plant and soybean mosaic virus (SMV) occurs wherever soybeans are grown, in part due to its seed transmissibility. Bao and colleagues developed a GFP-recombinant SMV system to assess SMV-soybean interactions [2]. The recombinant expressed GFP and induced symptoms in soybean plants and was effectively seed transmitted, thus allowing for a good system to assess SMV determinants of pathogenicity in soybeans, but also for SMV seed transmission. The latter serves as an important source of SMV primary inoculum in soybeans. They also created mutants and identified specific amino acids in the FRNK motif of the SMV HC-Pro that affected symptom development and oligomerization of HC-Pro, the multi-functional protein involved in aphid transmission and suppression of the plant RNAi anti-viral response.

Della Bartola and colleagues [3], and de Nazare Almeida Dos Reis and colleagues [4] used contemporary sequence and bioinformatics approaches to look at etiology and diversity of viruses in potatoes and tomatoes, respectively. Nanopore sequencing was used to identify complete genomes for four different viruses infecting potatoes, and even proved to be an effective approach for identification of potato virus Y variants and recombinants in mixed infections [3]. This practical application of this relatively inexpensive and portable technology provides a new platform for rapid, accurate identification of specific virus genotypes. Illumina HiSeq and bioinformatics were used to identify a complex of ssDNA viruses in field-grown tomatoes having and lacking the *Ty*-1 resistance gene, from different growing regions in Brazil [4]. New ssDNA viruses and an alpha satellite were discovered and as expected, more were found from tomatoes lacking the *Ty*-1 gene. However, one newly described begomovirus was recovered from tomatoes which had the *Ty*-1 resistance gene, suggesting the possibility of begomovirus adaptation to this widely used source of begomovirus resistance in tomatoes.

Three groups addressed the roles of non-coding RNAs in plant virus infections [5,6,7]. Liu and colleagues used microarray analysis to screen microRNAs (the microRNAome) and obtained a global profile allowing for the identification of some miRNAs and their roles as well as their corresponding target mRNAs, and resulting defense signaling pathways in beet necrotic yellow vein virus pathogenesis in *Nicotiana benthamiana* plants [6]. They also identified 8 *N. benthamiana*-specific miRNAs. Du and colleagues used heterologous viruses and short tandem target mimic (STTM) technology to assess the role of microRNA nbe-miR1919c-5p in infections of *N. benthamiana* plants by tobacco curly shoot virus (TbCSV) and its betasatellite (TbCSB) DNA [5]. Suppression of nbe-miR1919c-5p by STTM resulted in increased titers for TbCSV and TbCSB, plus more severe symptoms, including leaf curling in plants. Zhang and colleagues assessed the network of differentially expressed long non-coding RNAs (lncRNAs, >200 nt) and mRNAs in rice plants infected with rice black-streaked dwarf virus (RBSDV) [7]. The biological roles of lncRNAs are largely still unknown. They found that 22 lncRNAs were differentially expressed in RBSDV-infected rice plants. Interestingly, this study also found that specific differentially expressed mRNAs correlated with some of the lncRNAs identified here. Developing and applying a new approach to screen and identify lncRNA–mRNA regulatory pairs was another outcome of this study.

These papers are representative of the approaches and opportunities that are available now to gain a better fundamental understanding of plant virus-induced pathogenesis. Obtaining a more fundamental understanding of plant-virus interactions is necessary if we are going to develop efficacious, environmentally sound approaches to control plant diseases caused by plant viruses. We now have fantastic new tools including NGS and bioinformatics approaches to look at virus diversity and host responses to virus infection at the transcriptome but also small and large non-coding RNA levels, not only in different plant genotypes but also in different plant tissues. However, the diversity of papers here also shows that we cannot ignore virus-host biology, and we must consider biology to help us accurately interpret and use what we are finding with modern technologies.
